# Complete genome sequence of *Caulobacter vibrioides* CB2A JS4038

**DOI:** 10.1128/mra.01001-25

**Published:** 2025-12-22

**Authors:** Beth Davenport, Ryan McLaughlin, Tony Liu, Steven J. Hallam

**Affiliations:** 1 Department of Microbiology & Immunology, University of British Columbia 198130, Vancouver, BC, Canada; 2 Genome Science and Technology Program, University of British Columbia 8166, Vancouver, BC, Canada; 3 Graduate Program in Bioinformatics, University of British Columbia 8166, Vancouver, BC, Canada; 4 ECOSCOPE Training Program, The University of British Columbia 8166, Vancouver, BC, Canada; 5 Bradshaw Research Initiative for Minerals and Mining (BRIMM), University of British Columbia 8166, Vancouver, BC, Canada; 6 Life Sciences Institute, University of British Columbia 8166, Vancouver, BC, Canada; California State University San Marcos, San Marcos, California, USA

**Keywords:** genome announcement

## Abstract

We report the complete genome sequence of *Caulobacter vibrioides* CB2A JS4038. This strain is a variant of *C. vibrioides* CB2A containing genotypic changes needed to host a plasmid-encoded S-layer display system. PacBio long-read sequencing and assembly provide a closed reference genome, constraining the chromosomal location of these changes.

## ANNOUNCEMENT

*C. vibrioides,* a heterotypic synonym of *C. crescentus*, is a gram-negative, non-pathogenic, genetically tractable model organism, well studied for its dimorphic cell cycle and proteinaceous surface layer (S-layer) (1). A plasmid system for displaying heterologous peptides using insertions into the S-layer gene *rsaA* has been developed (2, 3). Here we report the complete genome sequence of *C. vibrioides* CB2A JS4038, a laboratory-derived variant (4) of *C. vibrioides* CB2A (GCA_003856435.1), which lacks S-layer expression due to a premature stop codon in *rsaA*. JS4038 carries additional modifications, including deletion of S-layer–associated protease (*sapA*) to prevent cleavage of fusion proteins, deletion of GDP-L-fucose synthase (*fcl*, required for EPS synthesis) to improve fusion protein interactions, and insertion of the *repBAC* operon for plasmid segregation and replication into the gene encoding 2-dehydro-3-deoxy-D-arabinonate dehydratase (*xylX*).

A glycerol stock of JS4038 stored at −80 °C was used to inoculate 5 ml of Peptone Yeast Extract (PYE) media (0.2% peptone, 0.1% yeast extract, 0.01% CaCl_2_, and 0.02% MgSO_4_) and grown for 48 h at 30 °C until reaching OD_600nm_ 0.8. Cells were pelleted at 4,000×g for 5 min, resuspended in 500 µL PYE, and transferred into a PowerBead Pro tube. Genomic DNA was extracted using the DNeasy PowerSoil Pro Kit (QIAGEN, 47014), yielding 23 ng/µL with an average fragment length of 23,000 bp, measured using a 4150 TapeStation (Agilent, G2992AA). DNA was sheared by pipette-tip cycling on a Microlab Nimbus (Hamilton), then SMRTbell libraries were prepared using PacBio SMRTbell Prep Kit 3.0 and size-selected on a BluePippin (3–50kb). Sequencing on the PacBio Revio platform using the Revio Polymerase Kit (HiFi chemistry) produced 755,900 reads totaling ~1.02 × 10¹⁰ bp, with an N50 of 14,801 bp and a maximum length of 51,091 bp. *De novo* assembly of raw reads using Flye (5) (v2.9.5-b1801) resolved a single 4,121,253 bp circular contig with a reported coverage of 121×, and an average GC content of 67.24%. Read and assembly statistics were calculated using seqkit (6) stat (v2.3.0) while coverage was self-reported by Flye. CheckM (7) (v1.2.2) assessment of the genome reported 98.70% completion and 0.00% redundancy. The NCBI Prokaryotic Genome Annotation Pipeline (8) (v6.10) was used to perform automated gene feature prediction and annotation (Table 1). Default parameters were used unless otherwise noted.

**TABLE 1:** Genome features of *Caulobacter vibroides* CB2A JS4038

Strain name	NCBI accession number	Genome size (bp)	GC (%)	Contigs	Genes	Protein CDS	Pseudogenes	rRNA operons	tRNA operons	Completeness/ contamination (%)
CB2A JS4038	CP189825	4,121,253	67.24	1	4,090	3,877	152	2	51	98.70/0.00

**Fig 1:**
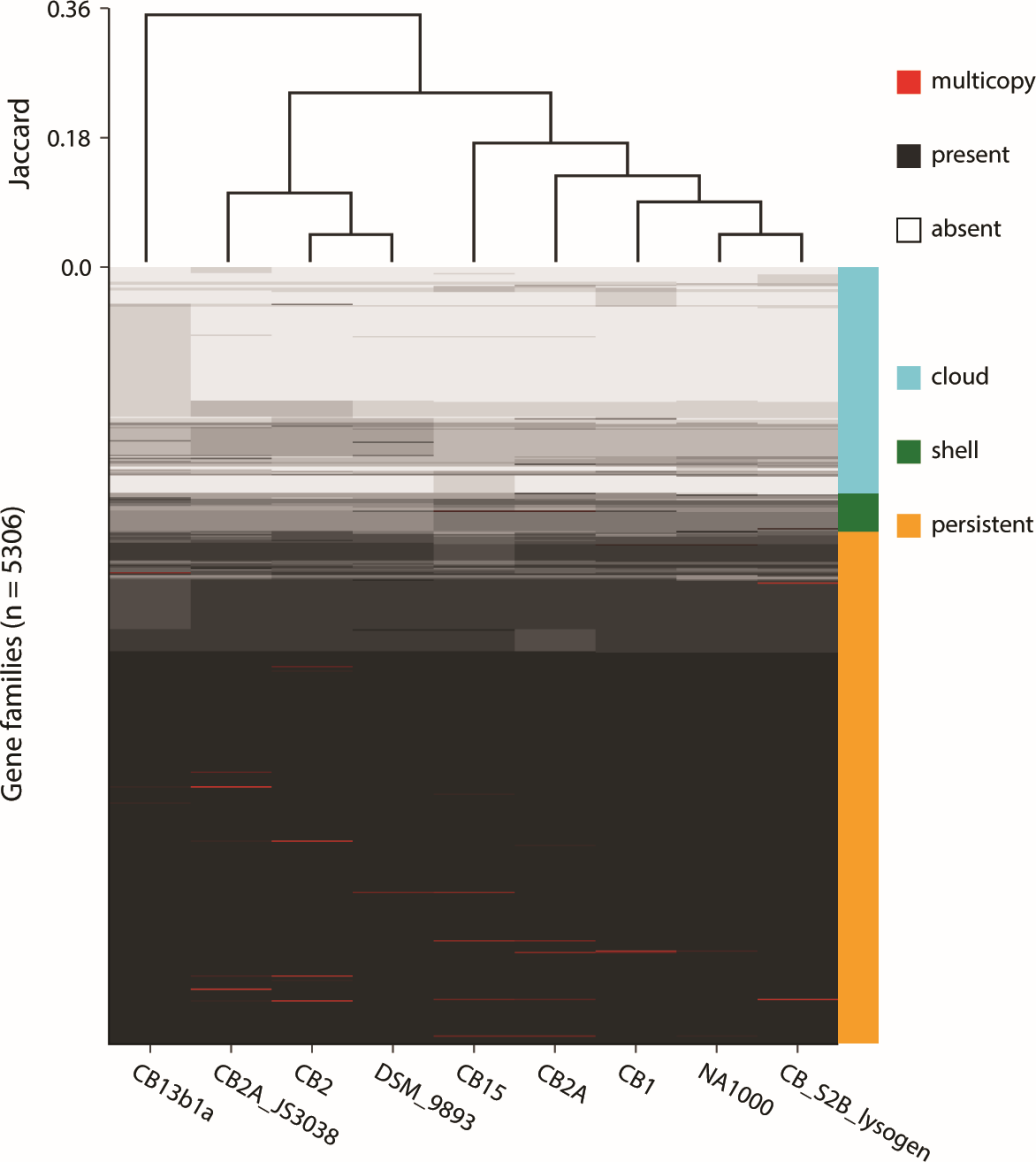
Presence-absence matrix of gene families across 8 *Caulobacter vibroides* genomes.

The resulting assembly and automated functional annotation resolved genotypic changes required for the S-layer display system, confirming the reported JS4038 genotype *ΔrsaA ΔsapA Δfcl ΔxylX::repBAC*. Specifically, *rsaA* (locus ACP82L_05345) contains a frameshift generating a stop codon at amino acid 360, *sapA* (locus ACP82L_03885) contains an internal deletion removing residues 108–449, *fcl* (locus ACP82L_02430) is truncated by loss of its first 75 amino acids, and *repBAC* insertion disrupts the native *xyl* promoter and removes the first 302 amino acids of *xylX* (locus ACP82L_04325). Comparative genomics of available *C. vibrioides* genomes using the PPanGGOLiN (9) pipeline (v2.2.4, default parameters; “all” and “draw” modules) (Fig. 1) showed CB2A shares greater gene content similarity with reference strains NA1000 (GCA_000022005.1) and CB15 (GCA_000006905.1) than with CB2 (GCA_002310295.2), while JS4038 clusters more closely with CB2, indicating the need to trace the lineage provenance further.

## Data Availability

The complete genome of *C. vibrioides* CB2A JS4038 is publicly available under NCBI accession no. CP189825, within BioProject accession no. PRJNA1255654 and BioSample accession no. SAMN48152127 (CB2A_JS4038, TaxID: 155892). Raw reads can be found in NCBI’s Sequence Read Archive (accession no. SRR35110076). Raw, intermediate, and final data products are stored on UBC’s Advanced Research Computing Chinook cloud storage resources, and computational steps are documented on GitHub (https://github.com/hallamlab/CB2A_genome_announcement).
